# Managing Diagnostic Uncertainty in Pediatric Sepsis Quality Improvement with a Two-Tiered Approach

**DOI:** 10.1097/pq9.0000000000000244

**Published:** 2020-01-11

**Authors:** Halden F. Scott, Allison Kempe, Sara J. Deakyne Davies, Paige Krack, Jan Leonard, Elise Rolison, Joan Mackenzie, Beth Wathen, Lalit Bajaj

**Affiliations:** From the * Department of Pediatrics, University of Colorado School of Medicine, Aurora, Colo.; † Department of Pediatrics, Section of Pediatric Emergency Medicine, Children’s Hospital Colorado, Aurora, Colo.; ‡ Adult and Child Consortium for Health Outcomes Research and Delivery Science, University of Colorado, Aurora, Colo.; § Department of Research Informatics, Research Informatics, Children’s Hospital Colorado, Aurora, Colo.; ¶ Center for Clinical Effectiveness, Children’s Hospital Colorado, Aurora, Colo.; ‖ Department of Pediatrics, Section of Pediatric Critical Care, Children’s Hospital Colorado, Aurora, Colo.

## Abstract

Severe sepsis requires timely, resource-intensive resuscitation, a challenge when a sepsis diagnosis is not confirmed. The overall goals were to create a pediatric sepsis program that provided high-quality critical care in severe sepsis (Sepsis Stat), and, in possible sepsis, flexible evaluation and treatment that promoted stewardship (Sepsis Yellow). The primary aims were to decrease time to antibiotics and the intensive care unit requirement.

**Methods:**

A 2-tiered clinical pathway was implemented at 6 pediatric emergency departments and urgent care centers, incorporating order sets, education, paging. The Sepsis Stat pathway included 2 nurses, hand delivery of antibiotics, resuscitation room use. The Sepsis Yellow pathway included prioritized orders, standardized procedures, close monitoring, and evaluation of whether antibiotics were warranted.

**Results:**

From April 2012 to December 2017, we treated 3,640 patients with suspected and confirmed sepsis. Among the 932 severe sepsis patients, the 30-day, in-hospital mortality was 0.9%. Arrival to recognition time improved from 50 to 4 minutes. Recognition to antibiotic time demonstrated an in-control process in our goal range with a median of 43 minutes for Sepsis Stat patients, 59 minutes for Sepsis Yellow patients. The proportion of severe sepsis patients requiring intensive care unit care declined from 45% to 34%. On the Sepsis Yellow pathway, 23% were de-escalated with discharge to home without antibiotics.

**Conclusions:**

This novel 2-tiered approach to pediatric sepsis quality improvement in varied emergency care settings improved process and outcome measures in severe sepsis while promoting stewardship and de-escalation where appropriate. Matching resources to the degree of illness was important in supporting quality care in potentially septic children.

## Abstract 2

Supplemental Digital Content is available in the text.

## INTRODUCTION

Severe sepsis affects >70,000 US children annually with pediatric mortality of 5%–20%.^1,2^ Recent medical, public, and governmental concern about the quality of sepsis care prompted laws in 2 states and a Centers for Disease Control campaign to improve pediatric sepsis care.^3–5^ The potentially life-saving first hour of pediatric sepsis care is guideline-concordant in <25% of cases.^6^ Timely diagnosis and treatment of pediatric sepsis can prevent organ failure, mortality, and morbidity.^7^

Severe sepsis is defined by organ dysfunction; however, experts recommend that treatment begin when a patient with suspected infection shows worrisome signs, often before confirmation of organ dysfunction.^8^ The diagnosis of early sepsis in children is difficult.^7,9^ While there is agreement that organ dysfunction with infection requires treatment, it is unclear how to identify patients who need treatment before organ dysfunction is evident.^10,11^ Under-diagnosing sepsis carries the risk of preventable mortality; however, routinely over-diagnosing sepsis incurs potential negative consequences of inappropriate antibiotic use and diverting resources from the care of other patients.^12^

Thus, this sepsis quality improvement (QI) program sought to balance rapid, resource-intensive resuscitation of children with severe sepsis, with diagnostic uncertainty and resource stewardship in the spectrum of febrile children presenting for emergency care.^13^ The QI team designed a clinical sepsis pathway with 2 severity tiers and promoted flexible escalation or de-escalation between the tiers. The overall goals were to create a program that provided high-quality critical care in severe sepsis (Sepsis Stat) and flexible, timely evaluation and treatment in possible sepsis that promoted stewardship (Sepsis Yellow). The primary aims were to decrease time to antibiotics and decrease the Intensive Care Unit (ICU) requirement through early resuscitation.

## METHODS

### Setting and Personnel

We formed an interdisciplinary QI team in 2012, with pediatric emergency and critical care physicians, nurses, and pharmacists, and stakeholders from inpatient and subspecialty services. The hospital’s Chief Quality Officer and Chief Medical Officer provided strong executive sponsorship of the program.

The setting was the Children’s Hospital Colorado Emergency Care Network, which included an academic, tertiary Emergency Department (ED) with >73,000 annual visits, and 5 satellite pediatric emergency care sites with ED, Urgent Care (UC), and ED/UC dual-track models with >100,000 annual visits among the 5 sites. A shared department of >150 providers, including pediatric emergency physicians, pediatricians, nurse practitioners, and physician assistants staffed all sites, with pediatric emergency nurses.

Sepsis QI began in 2012 and is described here through 2017. Children’s Hospital Colorado participated in the Children’s Hospital Association Improving Pediatric Sepsis Outcomes (IPSO) Collaboratives in 2012 and 2016 to the present, which provided frameworks for improvement and opportunities for learning from other institutions.

### Intervention

**Fig. 1.**
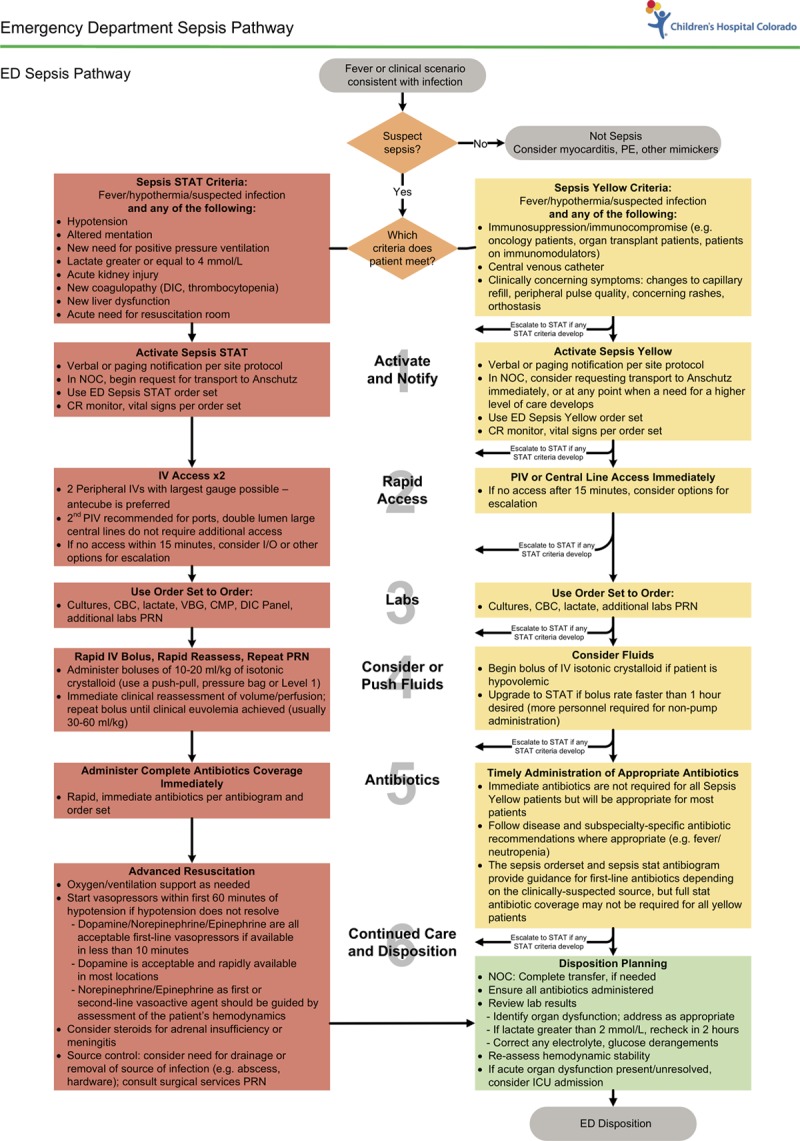
Two-tiered sepsis pathway.

The intervention was a 2-tiered clinical pathway for emergency pediatric sepsis care in patients >60 days (Fig. 1). The lower-severity tier would provide a pathway to address diagnostic uncertainty early in sepsis, by allowing clinicians to activate a flexible pathway when sepsis was not yet proven, and a more intensive, fixed pathway in cases of definitive severe sepsis. Patients with severe sepsis, organ dysfunction due to suspected infection, were treated on the Sepsis Stat tier. Patients in whom clinicians were concerned for potential sepsis without evident organ dysfunction were treated on the Sepsis Yellow tier.

We based the diagnostic criteria for inclusion in each pathway in Figure 1 on the American College of Critical Care Medicine guidelines.^13^ These definitions meant that some organ dysfunction could be diagnosed clinically, such as hypotension, respiratory dysfunction, or altered mental status, while others, such as hematologic dysfunction and acute kidney injury, required laboratory results. Clinicians were taught to activate the Stat pathway for any child with a critical illness or clinical organ dysfunction criteria and to escalate to the Stat pathway if a laboratory result returned indicating new organ dysfunction that had been previously unknown.

The Sepsis Yellow pathway included patients who did not demonstrate organ dysfunction: all immunocompromised children, those with a central line and fever, and any other patients in whom clinicians were concerned for sepsis based on history or examination findings. Although Sepsis Yellow patients did not yet demonstrate organ dysfunction, they nonetheless required early, expedited care for infection to prevent progression to severe sepsis. The QI team sought to address the patient who *might* have sepsis, in whom a diagnosis might become clear over the first hour of ED care without forcing a fixed, resource-intensive treatment on these possible sepsis patients.

There was not a universal screening tool that was used to identify patients with sepsis. Because none had been published at the start of this QI effort, this program focused on improving diagnosis through the use of the 2-tiered system, education, and feedback, with ongoing monitoring of diagnostic accuracy. A hypotension clinical decision support alert was introduced to supplement the diagnosis of shock from all etiologies by triggering escalation to the attending physician at the first occurrence of systolic hypotension (see Supplemental Digital Content at http://links.lww.com/PQ9/A152 for Figure 1).

The shared basic steps of care were the same: notification, intravenous (IV) access, laboratory studies, fluids, antibiotics, and resuscitation/disposition. A second nurse supported the more intensive therapies administered through the Stat pathway that included hand delivery of empiric antibiotics by a pharmacist, resuscitation room use, and ICU notification. The Sepsis Yellow pathway brought prioritized orders, enhanced monitoring, and standardized procedures. The Sepsis Stat pathway began in April 2012; the Sepsis Yellow pathway began in November 2012. Both pathways began in the community sites in June of 2013 (see Supplemental Digital Content at http://links.lww.com/PQ9/A152 for Table 1).

Both sepsis pathways were supported by order sets, paging, and education. Provider, nursing, and pharmacy education included an online module, in-person presentations at meetings, education at daily pre-shift nursing huddles, and individual audit and feedback. We provided audit and feedback in a letter to attending and fellow providers for every patient with severe sepsis; a sample letter is included in Figure 3 (Supplemental Digital Content at http://links.lww.com/PQ9/A152). We administered an internally designed online education module to nursing and provider staff, reviewing sepsis knowledge and local pathways.

### Data and Measures

Data about care and outcomes were extracted from the Epic Clarity database, transferred and loaded into REDCap, a secure, HIPAA-compliant web-based database. Patients in the database included all patients with the sepsis order set used or a sepsis page sent. We identified and included missed severe sepsis patients admitted to the ICU within 24 hours of ED care through a standardized chart review by 2 trained QI personnel. Patients who were excluded from analysis were <60 days of age, received antibiotics or a bolus before arrival, left without being seen, arrived with cardiopulmonary resuscitation in progress, arrived with an active “Do Not Resuscitate” order, or transferred to an external hospital. Because sepsis treatment was a criterion for inclusion into the registry, and comparable pre-intervention patients could not be reliably ascertained, data collection began in April 2012 at the start of overall sepsis QI, and October 2012 was the start of the 2-tiered system. We assessed the quality of care according to how patients were initially identified by clinicians, as well as by their final diagnosis of severe sepsis based on retrospective ascertainment of organ dysfunction.

The primary therapeutic process measure was time from recognition to antibiotic administration, intending to have a median time <54–102 minutes, the range reported as associated with improved outcomes in pediatric severe sepsis in peer-reviewed literature.^14–16^ We measured time from recognition to bolus, with a goal of <30 minutes in Sepsis Stat.^8^ The outcome measures were decreasing ICU admission in the first 24 hours of care and 30-day, in-hospital mortality. At a single institution, we did not expect to be able to see a significant mortality difference, so we set ICU admission as a primary outcome measure. We tracked outcome measures in all patients with severe sepsis, as defined by Goldstein et al.^13^

There were 2 diagnostic process measures: activation of the Sepsis Stat pathway among patients with severe sepsis and time from ED arrival to sepsis recognition. The pathway could be activated through the use of the paging system (used only at the 2 largest sites) or the Sepsis Stat order set. The goal was to increase the appropriate pathway use. Recognition time was the sepsis page or order set activation time, whichever was earliest, or time of IV antibiotic order if a page or order set was not used.

The pathway emphasized de-escalation when appropriate, based on the belief that activation of the pathway should begin before confirmation of severe sepsis, requiring the option to de-escalate if the initial suspicion for sepsis was incorrect. Thus, the balancing measure, called “safe de-escalation,” was the proportion of patients initially activated on a sepsis pathway who were discharged directly from the ED without ever receiving antibiotics and without revisit or death. We examined disposition categories to understand whether patients starting on 1 tier were escalated or de-escalated as their status changed.

We compared patient characteristics and hospital course characteristics for Stat and Yellow pathways using Wilcoxon and chi-squared tests, and also described characteristics of missed patients. Statistical Process Control charts were created in Minitab 17 (Minitab Inc, State College, Pa.) to evaluate the process measures. Nelson’s Rules were used to determine special cause variation, which is a method used to determine whether a change seen is likely due to chance alone or a change in the system.^17^

The Children’s Hospital Colorado Operational Quality Improvement Board approved data gathering for QI; Colorado Multiple Institutions Review Board approved the use of de-identified data for publication.

## RESULTS

From April 1, 2012, to December 31, 2017, 3,843 sepsis patients were treated. Nine hundred thirty-two patients had severe sepsis per consensus definitions for acute organ dysfunction and suspected infection in the ED.^13^ There was a substantial increase in the use of the sepsis system after the Sepsis Yellow tier was introduced (see Supplemental Digital Content at http://links.lww.com/PQ9/A152 for Figure 3). Patient characteristics are described in Table 1. Of the study population, 2,821 (73.4%) patients had chronic complex conditions as defined by ICD-10 codes,^18^ and 3,380 (88.0%) presented initially to the tertiary ED site. Patients treated on the Sepsis Stat pathway had more severe illness upon arrival and more severe hospital outcomes, including an ICU requirement and prolonged hospital length of stay (Table 1).

**Table 1.**
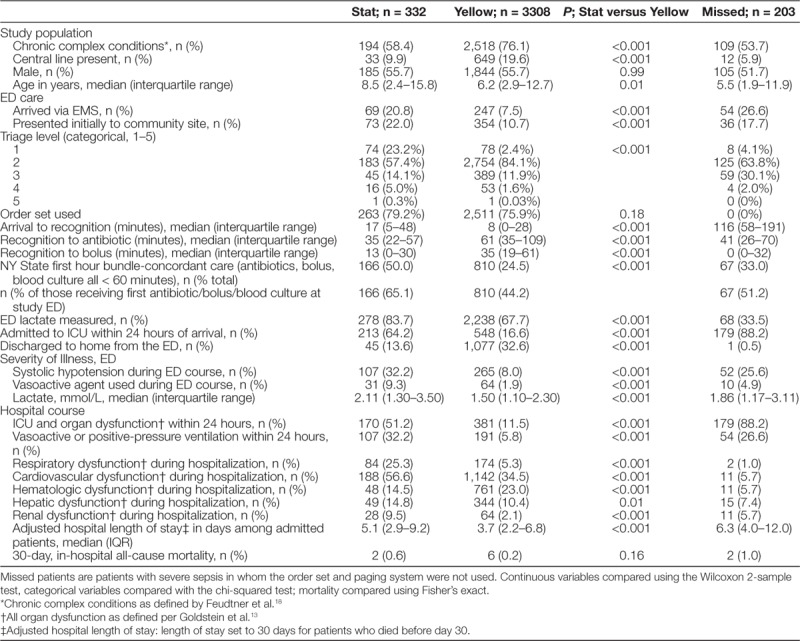
Study Population During All 5 Years of Quality Improvement, Compared by the Initial Tier of Clinical Sepsis Activation (Stat or Yellow)

**Fig. 2.**
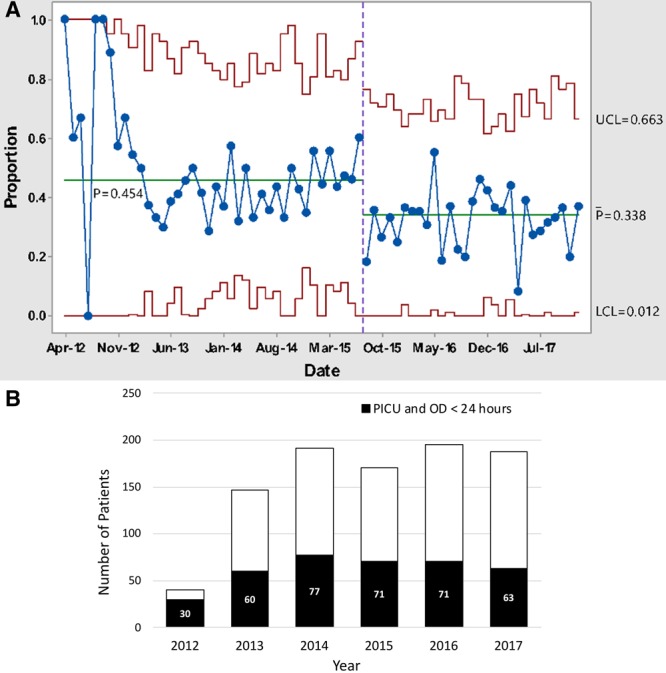
Outcome measure, ICU admission among severe sepsis patients. A, The proportion of severe sepsis patients requiring ICU care in the first 24 hours. B, The same severe sepsis patients, shown as absolute numbers by year. The height of the entire bar represents the number of patients with severe sepsis with acute organ dysfunction in the ED yearly; black bars represent the number of these requiring ICU care within the first 24 hours by year.

Our process measure, recognition to antibiotic time, was within the target range. The Statistical Process Control analysis demonstrated median recognition time to antibiotics was 42.8 minutes for patients on the Sepsis Stat pathway, faster than the goal of 54–102 minutes, with an in-control process (see Supplemental Digital Content at http://links.lww.com/PQ9/A152 for Figure 4). Patients on the Sepsis Yellow pathway were within goal range at 58.6 minutes. Our primary outcome measure, the proportion of severe sepsis patients who received ICU care after ED treatment, declined (Fig. 2A). This proportionate decrease was not driven by an increase in severe sepsis; absolute numbers of severe sepsis patients stayed constant (Fig. 2b).

**Fig. 3.**
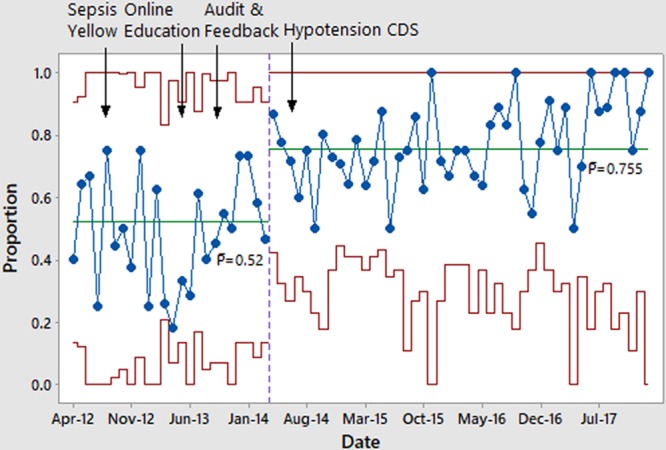
The proportion of patients in whom the Sepsis Stat system was appropriately activated each month, among patients meeting Sepsis Stat criteria.

**Fig. 4.**
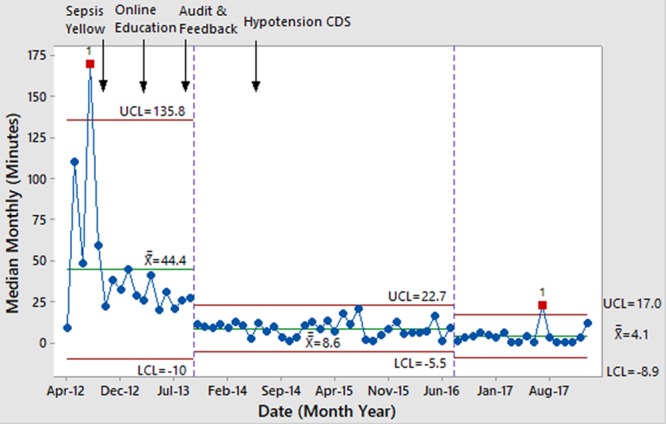
Time from ED arrival to sepsis recognition in patients with severe sepsis.

The diagnostic process measure of appropriate activation of sepsis stat improved over time (Fig. 3). The process measure of arrival to recognition improved, meeting criteria for special cause variation multiple times and showing decreased variation (Fig. 4). Notably, the introduction of the Sepsis Yellow tier was associated with one of these significant improvements. Recognition to bolus time was faster in Sepsis Stat patients, and was stable within goal range over time, without special cause variation (see Supplemental Digital Content at http://links.lww.com/PQ9/A152 for Figure 5).

The 30-day, in-hospital mortality rate among patients with severe sepsis was 0.9%, representing 8 patients. All patients who died were chronically ill; there were no deaths in previously healthy children. No 30-day mortalities occurred in sepsis registry patients who were discharged from the ED.

We evaluated the balancing measure of safe de-escalation (discharge home without IV antibiotics) by the initial treatment tier. A total of 794 patients initially suspected of sepsis had safe de-escalation, representing 23% of Sepsis Yellow and 10% of Sepsis Stat patients. The predominant dispositions of Sepsis Stat and Sepsis Yellow patients aligned with the severity of illness intended for each path: 64% of Stat patients were admitted to the ICU, and 60% of Sepsis Yellow patients were admitted to the ward.

## DISCUSSION

This QI program for pediatric sepsis was novel in several ways: it encompassed a tertiary ED and lower-acuity community-based ED/UC sites and included pediatric emergency physicians, general pediatricians, and nurse practitioners. The most important innovation of the program was using a 2-tiered approach to sepsis to address diagnostic uncertainty and resource utilization.

In keeping with their intent, the 2 tiers facilitated more rapid resuscitation in Sepsis Stat patients; and less use of antibiotics, laboratory tests, and hospitalization in Sepsis Yellow patients. We attained these results with a mortality rate of 0.9% among severe sepsis patients, lower than the reported mortality rates for single-tier pediatric sepsis ED protocols, which have reported pediatric severe sepsis mortality of 1.7%–3.9%.^14,15,19–21^ The proportion of patients receiving severe sepsis care in the ED who were admitted to the ICU within the first 24 hours was 34%. This outcome compares favorably with prior published descriptions of 39%–100% admission rates in similar pediatric severe sepsis populations.^15,19,20^

The median time from severe sepsis recognition to antibiotic administration of 42 minutes was among the fastest reported in pediatric sepsis literature.^6,15,20–22^ Time to recognition, a cognitive process, continued to improve over many years, while the time from recognition to antibiotic stabilized and no further improvement occurred. This finding may be because the nature of antibiotic delivery involves many physical tasks that are difficult to expedite beyond a certain point (securing venous access, sterile medication preparation, and physical movement to the patient’s bedside) and cognitive tasks (pharmacist medication review, pump programming) that cannot safely be expedited. The time to antibiotics on the Yellow pathway was longer, 58 minutes, and fewer resources were used to deliver antibiotics to these less acutely ill patients. While there is not a standard for time to antibiotics in patients with infection without organ dysfunction, mortality increases in severe sepsis when antibiotic administration is longer than 3 hours, so both pathways were well within this standard.^12,23^

The overall number of ED patients with severe sepsis remained constant over time, despite an increase in overall ED patient volume. Early treatment through the Yellow pathway may have decreased the proportion of patients ever meeting severe sepsis criteria.

The diagnostic approach of this pathway differs from previously described pediatric sepsis programs. When the program began, no sepsis screening tool had been tested and was not part of sepsis guidelines. Currently, there are no sepsis screening tools that have been externally validated or used in non-tertiary emergency or urgent care sites such as those in our system. Thus, we monitored the clinical diagnostic accuracy closely and found that it matched or exceeded screening tools later described by Cruz et al^22^ and Balamuth et al.^,24,25^ The 2-tiered system, as well as audit and feedback, likely facilitated clinical diagnostic accuracy. Measuring internal diagnostic accuracy is recommended by the American College of Critical Care Medicine (ACCM) pediatric sepsis guidelines “Measurement Bundle,” and was a critical component of this QI program.^8^ Tracking diagnostic accuracy and adjusting to any concerns should be an important component of any pediatric sepsis program, particularly one which promotes flexibility and de-escalation, such as this 2-tiered system.

Having 2 tiers of sepsis treatment increased the complexity of the system. When we first introduced the Sepsis Yellow pathway, there was a decrease in the appropriate use of the stat pathway, but improvement in recognition time. A lower resource-utilization tier may have encouraged clinicians to activate earlier, but also led to hesitancy to use the Sepsis Stat pathway. We noted an improvement in appropriate Sepsis Stat use after the introduction of audit and feedback (Fig. 3).

After this program began, several pediatric sepsis care bundles were described. The ACCM pediatric sepsis guidelines, the IPSO collaborative, and New York state first-hour bundle, differ from each other slightly, but share an emphasis on timely diagnosis, antibiotics, and bolus fluid.^6,8^ While we did not include or test every element of these bundles, we did find that care was concordant with the New York state bundle in 65% of patients in our Sepsis Stat pathway, exceeding bundle concordance reported in New York state (Table 1).^6^

We focused the Stat pathway on organ dysfunction without publishing age-specific abnormals for each category. This approach allowed the pathway to remain current when there were small changes in definitions in the medical literature and prevented the hospital system from having multiple definitions of abnormal. For example, although the ACCM guidelines have definitions for hypotension, the hospital followed PALS definitions for hypotension, which differed minimally in some age categories.^13^ The sepsis pathway maintained consistency with the PALS definitions, which already displayed as abnormal, allowing alignment with institutional education and EHR.

There were limitations to the assessment of this QI program. A complete pre/post comparison was not possible because we could not identify equivalent patients before the sepsis pathway began; the act of initiating sepsis QI led to increased sepsis evaluations and diagnoses. An additional limitation is the lack of certainty of the ultimate outcomes for discharged patients. We cannot determine for certain if a patient who was not in the sepsis registry later presented elsewhere with sepsis.

In conclusion, this pediatric sepsis QI program encompassed >3,500 patients. The severe sepsis pathway achieved process and outcome measures previously demonstrated in single-tier pathways, while the novel intermediate pathway demonstrated expedited early care with fewer personnel, laboratory tests, and medications. It achieved the 2 overarching goals: providing high-quality sepsis resuscitation to severe sepsis patients and promoting timely evaluation and treatment in possible sepsis patients. Flexibility and responsiveness were demonstrated, with escalation and de-escalation supported to promote resource and antibiotic stewardship. Matching resources to the degree of illness, and planning for de-escalation are important components of QI. In this case, tiered care was effective in addressing the clinical problem of early differentiation of potentially septic children.

## ACKNOWLEDGMENTS

The authors gratefully acknowledge the Children’s Hospital Association’s Improving Pediatric Sepsis Outcomes collaborative, a multi-hospital multiyear collaborative which provided centralized resources to support pediatric sepsis quality improvement work from 2016 onward.

## DISCLOSURE

The authors have no financial interest to declare in relation to the content of this article.

## Supplementary Material

SUPPLEMENTARY MATERIAL
