# Supplementary figures and images for: Managing Diagnostic Uncertainty in Pediatric Sepsis Quality Improvement with a Two-Tiered Approach
Source: Pediatr Qual Saf. 2020 Jan 11;5(1):e244. doi: 10.1097/pq9.0000000000000244 (PMC7056288; doi:10.1097/pq9.0000000000000244)

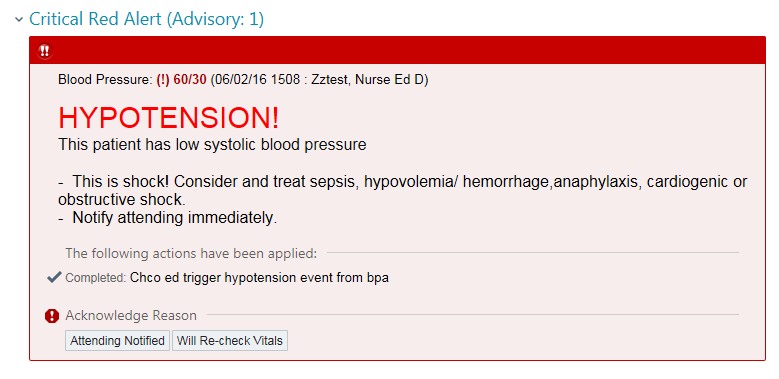

Supplement: SUPPLEMENTARY MATERIAL [file pqs-5-e244-s001.tiff]

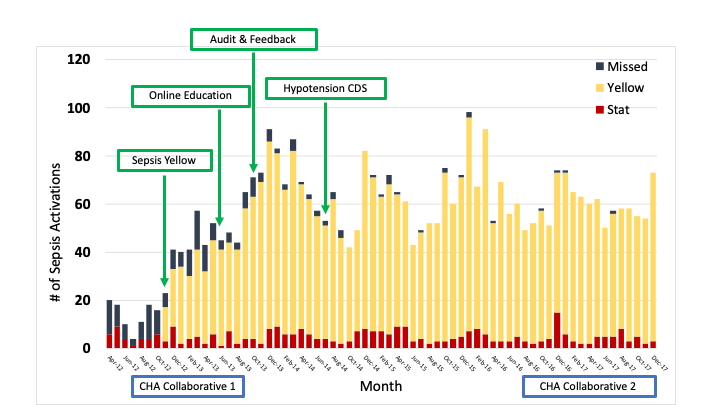

Supplement: SUPPLEMENTARY MATERIAL [file pqs-5-e244-s003.tiff]
