# Supplementary material for: Managing Diagnostic Uncertainty in Pediatric Sepsis Quality Improvement with a Two-Tiered Approach
Source: Pediatr Qual Saf. 2020 Jan 11;5(1):e244. doi: 10.1097/pq9.0000000000000244 (PMC7056288; doi:10.1097/pq9.0000000000000244)
Supplement: SUPPLEMENTARY MATERIAL [file pqs-5-e244-s004.docx]

Table, Supplemental Digital Content 2. Adaptations of quality improvement elements at the six sites

| Quality Improvement Element | Fidelity/Adaptation in Implementation |
| --- | --- |
| Core Elements Implemented Consistently Across Sites | |
| Diagnostic criteria | Core Element, no variation across sites |
| STAT / Yellow pathway | Core Element, no variation across sites |
| Order Sets | Core Element, no variation across sites |
| Antibiotic choice | Core Element, no variation across sites |
| Fluid resuscitation strategy | Core Element, no variation across sites |
| Elements Adapted to Site-Specific Needs | |
| Notification of sepsis activation | Adapted. Pagers used at larger sites, verbal face-to-face notification at smaller sites. |
| Antibiotic preparation | Adapted. Prepared by nurses at sites without on-site pharmacists, and by pharmacists when pharmacist on-site. Pharmacy staffing at the smaller sites varied by site, year, and time of day. |
| Laboratory testing | Adapted. Bedside point-of-care testing used at smaller, community sites without full laboratory on-site. Labs universally available included: lactate, complete blood count, basic metabolic panel and blood gas. Additional labs were run on-site at the larger sites and sent via courier to main hospital at smaller sites. Many patients requiring additional labs were themselves also transferred prior to additional lab results being known. |
| Disposition | Adapted. Patients requiring admission were transferred to one of three sites with inpatient units. Patients with actual or potential organ dysfunction emergently transferred to the tertiary hospital with an ICU. |
