# Supplementary material for: Managing Diagnostic Uncertainty in Pediatric Sepsis Quality Improvement with a Two-Tiered Approach
Source: Pediatr Qual Saf. 2020 Jan 11;5(1):e244. doi: 10.1097/pq9.0000000000000244 (PMC7056288; doi:10.1097/pq9.0000000000000244)
Supplement: SUPPLEMENTARY MATERIAL [file pqs-5-e244-s002.docx]

June 20, 2017

Dear Dr. [ED Attending & Fellow]:

Congratulations on the outstanding sepsis care which you delivered to your patient! You met 3/3 of our quality metrics. [Delete if they did not]

Last month, you provided care to a patient in the emergency department who met criteria for sepsis stat activation. As part of our efforts to continue to improve the quality of care that we deliver to patients with sepsis, we are providing monthly feedback on all patients who met Sepsis Stat criteria. This is only for your own improvement – we are not tracking statistics on providers!

As a reminder, **Sepsis Stat** is appropriate for any patient with **suspected infection plus critical illness** (hypotension, lactate ≥4.0 mmol/L, severely altered mental status, or severe respiratory distress requiring ventilation above baseline). Every month, our ED sepsis team reviews sepsis activations to determine whether a patient met Stat criteria, and reviews these cases in depth.

Our metrics align with national consensus metrics. Some groups consider recognition time “Time 0” and some consider arrival to be “Time 0.” We provide information on both. “Recognition-to” times are a bare minimum goal. Striving for appropriate arrival to recognition is critical, but it is difficult to set an automated system-wide goal, so you should review your own case and decide if arrival to recognition time was appropriate in your case. Sepsis orderset use or page is considered “recognition,” if you did not use the sepsis system, then time of antibiotic order is considered recognition, followed by triage if no antibiotics were ordered.

**MRN:	Date of Visit:**

|  | **Target** | **Your Case** | **Achieved Goal?** |
| --- | --- | --- | --- |
| **Type of Activation** | **Stat** | **Stat** | **Yes** |
| **Arrival to recognition time** | **N/A** | **8 min** | **N/A** |
| **Recognition-to-bolus time** | **≤30 min** | **18 min** | **Yes** |
| **Recognition-to-antibiotic time** | **≤60 min** | **37 min** | **Yes** |
| Additional comments from sepsis team review: | | | |

As always, please contact us to discuss this case, or any aspect of the sepsis program. Thank you for your contribution to providing outstanding care to children with sepsis, and all children, in our emergency department.

Sincerely,

Halden Scott, MD	Joni Mackenzie, MS, RN, CPNP, CPEN

Physician Lead, CHCO Sepsis Initiative	ED Nursing Lead, CHCO Sepsis Initiative
